# The Rare Malignancy of the Hepatobiliary System: Ampullary Carcinoid Tumor

**DOI:** 10.1155/2011/173036

**Published:** 2011-05-31

**Authors:** Mustafa Ozsoy, Yucel Ozsoy, Aras Emre Canda, Olcay Ak Nalbant, Fatih Haskaraca

**Affiliations:** ^1^Department of General Surgery, Manisa State Hospital, 45000 Manisa, Turkey; ^2^Department of General Surgery, Dokuz Eylul University School of Medicine, Izmir, Turkey; ^3^Department of Pathology, Manisa State Hospital, Manisa, Turkey; ^4^Department of General Surgery, Merkezefendi State Hospital, Manisa, Turkey

## Abstract

*Introduction*. Carcinoid tumors are low-grade tumors originating from endoderm and mostly involving the gastrointestinal system. However; they may be seen in any site within the gastrointestinal system. *Case Presentation*. A 69-year-old female patient. The results of blood tests were observed to be consistent with obstructive jaundice. A mass appearance was not encountered on tomographic examination. Papilla that was tumor-like macroscopically was seen in the second part of the duodenum in diagnostic endoscopy. Pylorus—preserving pancreaticoduodenectomy surgical procedure was applied. On pathological examination of the mass, a tumoral mass was detected in ampulla vateri localization, 1.5 × 1 × 0.8 cm in size, which, in immunohistochemical staining, was evaluated as a neuroendocrine tumor. Also, Metastasis was observed. *Conclusion*. The rarest type of carcinoid tumor is ampullary located carcinoid tumor, and tumor size is not a reliable indicator for tumor aggressivity in ampullary carcinoid tumors.

## 1. Introduction

Carcinoid tumors are low-grade tumors originating from endoderm and mostly the involving gastrointestinal system. Although they may be seen at any site in the whole gastrointestinal system, the most commonly involved areas are appendix, distal small intestine, rectum, and stomach [1]. Herein, we described the rarest type of carcinoid tumor. 

## 2. Case Report

**Figure 1:**
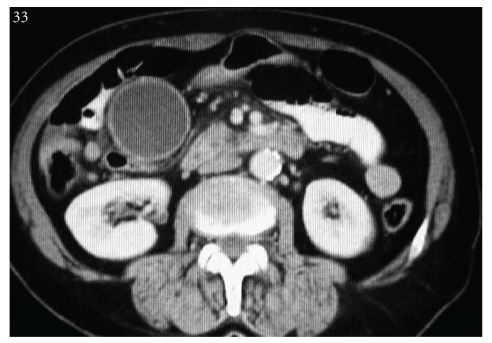
Hydropic gallbladder and choledoc dilation were seen on tomographic examination, Yet any mass appearance or lymph node was not encountered.

**Figure 2:**
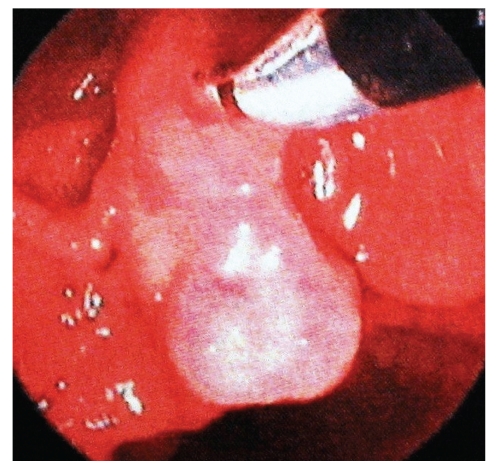
Papilla that was tumor-like macroscopically was seen in endoscopic retrograde cholangio pancreatography.

**Figure 3:**
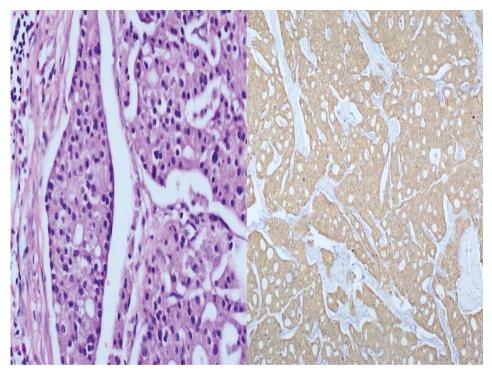
Specimens were stained synaptophysin (+), chromogranin-A (+), Ki–67 2% (+) in immunohistochemical staining.

A 69-year-old female patient was examined as jaundice was added to abdominal pain, accompanied by intermittent emesis and vomiting, lasting for 3 months. Results of blood tests were observed to be consistent with obstructive jaundice. A mass appearance was not encountered on tomographic examination obtained with prediagnosis of pancreatic cancer due to hydropic gallbladder and choledoc dilation on abdominal ultrasonography (Figure 1). Papilla that was tumor-like macroscopically was seen in the second part of the duodenum in diagnostic endoscopy (Figure 2). The patient, whose endoscopic biopsy result was evaluated as a well-differentiated endocrine tumor, was operated on. An ovoid mass 2 × 2 cm in size was detected in ampulla vateri in intra-abdominal exploration. Pylorus—preserving pancreaticoduodenectomy surgical procedure was applied to the patient as the mass did not make vascular invasion but local invasion. The patient in whom a major surgical complication was not observed in postoperative followup was discharged in the postoperative second week. On pathologic examination of the mass, a tumoral mass was detected in ampulla vateri localization, 1.5 × 1 × 0.8 cm in size, and invaded submucosa and muscularis propria. The patient whose specimens were stained synaptophysin (+), chromogranin-A (+), and Ki-67 2% (+) in immunohistochemical staining was evaluated as a neuroendocrine tumor (Figure 3). Metastasis was observed in one out of 10 lymph nodes that were sorted out in the peripancreatic region of the patient whose surgical margins were healthy. No other foci were encountered in octreotide scintigraphy in the postoperative first month in the patient who was diagnosed as a well-differentiated endocrins tumor on histopathologic evaluation. Adjuvant chemotherapy and biologic treatment were not planned because of the absence of carcinoid syndrome findings and poor prognostic histopathologic properties. 

## 3. Discussion

Ampullary located carcinoid tumor was first described by Oberndorfer in 1907 [2]. Although most of the reports are comprised of case reports, approximately 120 cases have been reported to date. It is rarer than even duodenal involvement defined as rare (2%) with this number (0.05%) [3, 4]. Its clinical presentation is composed of jaundice (53,1%), abdominal pain (24,6%), pancreatitis (6,0%), and weight loss (3,6%) [1–4]. Abdominal tomography and endoscopic imaging methods are also valuable besides clinical findings for making a diagnosis. Of endoscopic imaging methods, especially *endoscopic* retrograde cholangio pancreatography, endoscopic biopsy and endosonographic ultrasonography may be used for the detection of invasion depth [5]. In 25% of ampullary carcinoid tumors, they are seen to be related with von Recklinghausen syndrome and MEN syndromes. Additionally, most of the ampullary tumors have been seen to be positive in terms of somatostatin in pathologic examination. Thus octreotide scintigraphy is used with the aim of screening [6]. For carcinoid tumors with unknown malignity potential, a novel classification system was developed by the World Health Organization which classes tumors as benign, potentially malignant and malignant. In this classification, tumor proliferative index, invasion depth, and anatomic location were evaluated [7, 8]. Ampullary carcinoid tumors still have obscurities about the issues like prognostic features, invasion depths, and lymph node metastasis because of its prevalence [9]. These obscurities also reflect on surgical treatment strategy of ampullary carcinoid tumors. Makhlouf et al. stated that metastatic disease was not in question for duodenal carcinoid tumors smaller than 20 mm, whereas the same may not be said for ampullary carcinoid tumors. In literature, the presence of metastatic disease was seen in 40–50% of ampullary carcinoid cases smaller than 20 mm. Thus it is advocated that the most appropriate surgical treatment option should be the Whipple procedure [9, 10].

In our case, metastasis of lymph node was found although the tumor size was below 20 mm, and all signs were not marked by the CT scan. Today, with the knowledge gained from oncologic surgical interventions, pancreaticoduodenectomy has become routinely practicable without any complications, which was inconceivable in the past. Therefore, we consider that, the most appropriate surgical treatment option for ampullary carcinoid tumors must be the Whipple procedure. Hwang et al. detected one-year survey as 90% and three-year survey as 64% in 10 patients who underwent pancreaticoduodenectomy with a mean tumor size of 2.1 ± 1.3 cm [11]. 

The opponent group states that lymph node dissection does not have a therapeutic effect on ampullary carcinoid tumors and thus recommends local excision or endoscopic resection with lower morbidity and mortality [12, 13]. However, as described by Clement et al., the possibility of residual tumoral formation is high with local resection, just as in the case that showed local recurrence 20 months after local excision [14]. These findings indicate that tumor size is not a reliable indicator for tumor aggressivity in ampullary carcinoid tumors. Adjuvant treatment options should be planned by taking prognostic factors of tumor and carcinoid syndrome findings into consideration. Adjuvant chemotherapy may be administered following resection in tumors that have poor prognostic factors. The most commonly used agents for chemotherapy are streptozocin, doxorubicin, dacarbazine, and 5-fluorouracil. Other treatment options are biologic therapy (interferon INF, somatostatin analogues SST) and chemoembolization [13, 14]. 

## 4. Conclusion

Gastrointestinal system-related deaths are placed highly among cancer-related deaths. Hepatobiliary system cancers are the most feared tumors among all gastrointestinal system tumors with a prevalence of 7–300/100.00 [15]. The only known potential therapeutic treatment option for these cancers is surgical RO resection. The presence of lymph node metastasis and tumor size is known as an independent prognostic factor for hepatobiliary cancers. However, tumor size is not a reliable indicator for tumor aggressivity in ampullary carcinoid tumors.
